# First-line treatment with oxaliplatin and capecitabine in patients with advanced or metastatic oesophageal cancer: a phase II study

**DOI:** 10.1038/sj.bjc.6603750

**Published:** 2007-04-17

**Authors:** E van Meerten, F A L M Eskens, E C van Gameren, L Doorn, A van der Gaast

**Affiliations:** 1 Department of Medical Oncology, Erasmus MC - University Medical Centre Rotterdam, The Netherlands

**Keywords:** oesophageal cancer, oxaliplatin, capecitabine

## Abstract

This phase II study assessed the safety and efficacy of oxaliplatin and capecitabine in patients with advanced oesophageal cancer. Fifty-one eligible patients received oxaliplatin 130 mg m^−2^ intravenously on day 1 and capecitabine 1000 mg m^−2^ orally twice daily on days 1 to 14 in a 21-day treatment cycle as first-line treatment for advanced oesophageal cancer. Grade 3 neutropenia was seen in one patient and anaemia in another patient. No grade 4 haematological toxicities were observed. Grade 4 non-haematological toxicity (lethargy) occurred in one patient (2%). Grade 3 non-haematological toxicity was seen in 14 (27%) patients (vomiting and polyneuropathy (8%); nausea (6%); lethargy and hand–foot syndrome (4%); and anorexia, diarrhoea, and hyperbilirubinaemia (each in one patient)). In 22% of the patients, toxicity was the reason for stopping the treatment. The overall response rate was 39%. The median overall survival was 8 months; the 1-year survival rate was 26%. In the quality of life (QoL) analysis, the emotional well-being improved during treatment, but the physical functioning scores declined. The fatigue score on the symptom scales increased. Overall, the global QoL score did not change during treatment. In conclusion, the activity of oxaliplatin and capecitabine is comparable with other chemotherapy regimens in advanced oesophageal cancer with a low frequency of grade 3/4 toxicity. Because this treatment can be given on an outpatient basis, it is probably less toxic than cisplatin-based therapy and preserves QoL during treatment, it is a viable treatment option in patients with advanced oesophageal cancer.

Patients with oesophageal cancer generally have a poor prognosis, because the majority of them already have locally unresectable or metastatic disease at presentation. Furthermore, even after surgery with curative intent, local recurrences and/or distant metastases are detected in approximately two-thirds of the patients within 5 years of follow-up (Hofstetter *et al*, 2002). Many patients with oesophageal cancer require palliative therapy to treat symptoms, such as dysphagia. Placement of a self-expanding metal stent, external beam radiotherapy, intraluminal radiotherapy (brachytherapy), and laser therapy are commonly used palliative modalities to treat dysphagia (Homs *et al*, 2005).

Palliative chemotherapy may result in local and distant tumour and symptom control. The effect of chemotherapy on survival is unclear, mainly owing to a lack of randomised trials. The most frequently used chemotherapy regimen for patients with metastatic disease is a combination of 5-fluorouracil and cisplatin, with response rates ranging from 15–45% (Ilson, 2003). However, treatment with 5-fluorouracil and cisplatin can induce severe toxicity (Bleiberg *et al*, 1997). Besides, most patients have to be hospitalised for this treatment. In two trials, a significant positive effect of chemotherapy on quality of life (QoL) and/or overall survival was demonstrated (Webb *et al*, 1997; Ross *et al*, 2002). However, in both trials, patients with oesophageal and gastric cancer (predominantly adenocarcinomas) were treated. There are no studies comparing the effect of chemotherapy and other palliative treatments on symptom control (e.g. dysphagia).

Capecitabine is a novel oral fluoropyrimidine carbamate, which is converted into 5-fluorouracil, preferentially in tumours. Clinical studies with capecitabine have been predominantly performed in colorectal and breast cancer. In a study performed by Hoff *et al* (2001) in patients with advanced colorectal cancer, treatment with capecitabine was at least as effective as treatment with 5-fluorouracil plus leucovorin, but leaded to less hospitalisations for adverse reactions. Swallowing tablets might be a problem in patients with oesophageal cancer. However, it is our experience that patients with grade 1 or 2 dysphagia are still able to ingest tablets. Patients with grade 3 or 4 dysphagia are usually first palliated with either a stent or brachytherapy and are thereafter nearly always able to ingest tablets.

The response rate of single-agent cisplatin in metastatic oesophageal cancer is 6–26% (Kok, 1997), whereas that of carboplatin is less than 10% (Sternberg *et al*, 1985; Mannell and Winters (1989)). Oxaliplatin is a third-generation platinum compound. It forms inter- and intrastrand cross-links with DNA. These cross-links inhibit DNA replication and transcription. It has demonstrated synergy with 5-fluorouracil in advanced colorectal cancer (de Gramont *et al*, 2000; Giacchetti *et al*, 2000).

The combination of oxaliplatin and capecitabine has been tested in several phase II studies in patients with metastatic colorectal cancer (Borner *et al*, 2002; Cassidy *et al*, 2004). Grade 3/4 diarrhoea was seen in 33–50% of the patients treated with capecitabine 1250 mg m^−2^ twice daily and oxaliplatin 130 mg m^−2^ in the study performed by Borner *et al* (2002). Cassidy *et al* (2004) reported grade 3/4 diarrhoea in 16% of the patients treated with oxaliplatin 130 mg m^−2^ and capecitabine 1000 mg m^−2^ twice daily. The response rate was comparable, 49 and 55%, respectively. Therefore, a capecitabine dose of 1000 mg m^−2^ twice daily on days 1 to 14 in combination with oxaliplatin 130 mg m^−2^ on day 1 in a 21-day treatment cycle is the recommended dose.

Based on these favourable results of oxaliplatin combined with capecitabine in other gastrointestinal malignancies, we conducted the present phase II study to evaluate the safety and efficacy of the combination of oxaliplatin and capecitabine in patients with metastatic or local–regional unresectable carcinoma of the oesophagus, oesophagogastric junction, and cardia. In addition, to evaluate the effects of this schedule on the patients' well-being, we performed a QoL analysis on these patients during the treatment.

## METHODS

### Eligibility criteria

Eligible patients had histologically proven metastatic or local–regional unresectable carcinoma of the oesophagus or gastric junction and at least one unidimensionally measurable lesion ⩾20 mm using conventional computed tomography (CT) or magnetic resonance imaging scan or ⩾10 mm using spiral CT scan had to be present. No prior chemotherapy for locally advanced or metastatic disease was allowed. Patients were required to be aged at least 18 years, to have an Eastern Cooperative Oncology Group performance status of ⩽2 and a life expectancy of ⩾3 months. Other criteria included adequate haematological, renal, and hepatic functions as defined by: granulocyte count of at least 1500 mm^−3^ and platelet count 100 000 mm^−3^; serum creatinine ⩽1.25 × the upper normal limit (ULN); aspartate aminotransferase and alanine aminotransferase ⩽3 × ULN (⩽5 × ULN in case of liver metastases) and bilirubin ⩽1.25 × ULN. Previous neoadjuvant treatment for non-metastatic disease was allowed if completed at least 6 months before the initiation of study treatment. No history of malignancy, apart from non-melanomatous skin cancer, curatively treated carcinoma *in situ* of the cervix, or a ‘cured’ malignancy more than 5 years before enrolment was allowed. Patients with evidence of central nervous system metastases, a lack of physical integrity of the upper gastrointestinal tract, a malabsorption syndrome, or an inability to take oral medication were excluded. Patients were not eligible if they had a pre-existing motor or sensory neurotoxicity > grade 1 according to the National Cancer Institute Common Toxicity Criteria (NCI-CTC version 3). The Ethics Committee at Erasmus University Medical Centre approved the study and written informed consent was obtained.

### Treatment

Treatment consisted of oxaliplatin 130 mg m^−2^ intravenously on day 1 and capecitabine 1000 mg m^−2^ orally twice daily on days 1–14 (28 doses), repeated every 3 weeks. Oxaliplatin diluted in 250 ml of 5% glucose was administered as a continuous infusion over 2 h. Capecitabine had to be ingested with water every 12 h, approximately 30 min after a meal, starting at the evening of day 1. Prophylactic anti-emetic use of a 5-HT 3 antagonist and dexamethason was given. All patients continued either with metoclopramide, domperidone, steroids, or 5-HT 3 antagonists.

Dose modifications were made for toxicity, using the NCI-CTC (version 3). The absolute neutrophil count and the platelet count had to be recovered to the required pre-treatment values before start of the next treatment cycle. Non-haematological toxicity had to be ⩽ grade 1 before start of every treatment cycle. If these conditions were not met, dosing was delayed for a maximum of 2 weeks. If haematological or non-haematological toxicity was not recovered to grade 1 or less after 2 weeks, patients were taken off-study.

Persistent (⩾14 days) paresthesia or temporary (7–14 days) painful paresthesia or functional impairment prompted a 25% dose reduction of oxaliplatin. In case of persistent (⩾14 days) painful paresthesia or functional impairment, oxaliplatin had to be omitted until recovery and had to be restarted at 50% of the dose. Patients went off-study if these toxicities recurred despite the dose reductions.

Capecitabine was reduced with 25% in case of grade 2 hand—foot syndrome. In case of grade 2–4 diarrhoea, capecitabine intake had to be interrupted immediately. Standard treatment for diarrhoea was prescribed (i.e. loperamide). The omitted doses were not permitted to be administered after resuming treatment and the total length of capecitabine treatment period was not allowed to exceed 14 days. If patients experienced severe capecitabine-related toxicity (>grade 2) despite two dose reductions, necessitating discontinuation of treatment with capecitabine, patients were taken off-study.

Patients who showed no disease progression and/or prohibitive toxicity continued treatment for six courses, with a maximum of eight courses in case of ongoing response.

### Pre-treatment and follow-up evaluation

Pre-treatment evaluation included a detailed history taking, a physical examination, and routine blood examinations. All patients underwent a baseline CT of the chest and upper abdomen. After discontinuation of treatment, follow-up visits were done every 3 months to document late toxic effects, disease progression, and survival.

### Evaluation of response and toxicity

Patients were evaluable for response after two courses of chemotherapy. Evaluation of response was done every other course of chemotherapy. However, if tumour progression was found at any time after randomisation, it was recorded as progressive disease. Tumour response was assessed according to the Response Evaluation Criteria In Solid Tumours (UICC, 2002). The duration of response was measured from the time of complete or partial response until the first date of recurrent or progressive disease. Stable disease was measured from the start of treatment until the criteria for progression were met. Progression-free and overall survival was documented from the time of patient randomisation until tumour progression or death.

### QoL assessment

Quality of life was measured using the EORTC QLQ-C30 (version 3.0) and QLQ-OES18 (Aaronson *et al*, 1993; Blazeby *et al*, 2003). Questionnaires were filled in before therapy, after every other cycle, and after completion of chemotherapy. Patients with missing forms were excluded from the analysis of the absent assessment point. Scores were calculated according to the guidelines, yielding a range of 0–100. A higher score for a functional scale represents a higher level of functioning. A higher score for a symptom scale/item represents a higher level of symptomatology/problems (Fayers *et al* (2001)). Because high dropout rates result in more favourable scores among the remaining patients, comparisons were only made between baseline and after the second course and between baseline and after stopping chemotherapy.

### Statistical analysis

An optimal two-stage design for phase II trials as described by Simon was used (Simon, 1989). In the first stage, a total of 13 patients were included and at least four responses were required to continue to the second stage. In the second stage, 30 additional patients were included to a total sample size of at least 43. Thirteen responses were needed to conclude with a 95% confidence that the response rate was greater than 40%. Statistical differences in QoL at different time points were determined using the *t*-test. All tests were two sided at the .05 level of significance. The SPSS statistical package (version 12.0; SPSS Inc., Chicago, IL, USA) was used for all statistical analyses.

## RESULTS

### Patient characteristics

From April 2003 to October 2005 51 patients were included in this study. The baseline characteristics are summarised in Table 1. The majority of the patients were male (86%) and most of them had adenocarcinoma (88%).

### Toxicity of and adherence to chemotherapy

The median number of treatment cycles was four (range 1–8). Apart from progression of disease or end of protocol, reasons for stopping chemotherapy were toxicity (22%), patient's request (4%), and clinical deterioration (2%). The dose of capecitabine was reduced in five patients (10%), either because of diarrhoea (*n*=3) or hand–foot syndrome (*n*=2). In four patients, the administration of capecitabine was prematurely interrupted because of diarrhoea. The dose of oxaliplatin was reduced in three patients because of painful paresthesia.

Haematological toxicity is summarised in Table 2. Apart from one case each of grade 3 neutropenia and grade 3 anaemia, no grade 3 or 4 haematological toxicities were observed. In the latter, patient analysis showed an undetectable haptoglobin and an increased LDH and bilirubin, indicating haemolysis, which has been described in relation to oxaliplatin administration (Taleghani *et al*, 2005).

Non-haematological toxicity is summarised in Table 2. Eighteen patients were hospitalised during treatment. In eight patients, this was directly related to the treatment (dehydration caused by anorexia, nausea, vomiting, and/or diarrhoea (*n*=6), grade 4 lethargy (*n*=1), observation for one night in hospital after laryngopharyngeal dysesthesia (*n*=1)). In two patients, the hospitalisation was possibly related to the treatment (venous thromboembolism). The other eight hospitalisations were because of disease-related problems, such as dysphagia requiring oesophageal stenting, jaundice, ileus, pericarditis, fever, and tumour-related bleeding.

### Response

Forty-nine patients were evaluable for response. No complete responses were seen. Nineteen patients (39%) achieved a partial response, 21 patients (43%) had stable disease, and nine patients (18%) had disease progression. The median duration of response was 5.3 months (range 2–18).

### Survival

All 51 patients were evaluable for survival. At the date of evaluation (April 15, 2006) 43 patients have died. The median survival time for all patients was 8 months (95% CI 6–9 months, range 2–27 months). The 1-year overall survival was 26% and 2-year overall survival was 7%.

### QoL

Hundred and forty-one of 165 expected questionnaires were completed (85%). Four patients were excluded because QoL data were not obtained before the start of treatment. The scores of the responders were not different from the scores of the non-responders.

From 41 patients we obtained the QLQ-C30 questionnaires at baseline and after the second course, from 39 patients the OES18 questionnaires at these time points. Although the physical functioning score declined significantly from 85 to 78 (*P*=0.04), the emotional functioning score improved significantly from 61 to 73 (*P*=0.003). The other functional scores did not change. The sleeping score of the EORTC QLQ-C30 decreased significantly from 34 to 16 (*P*=0.006), indicating improvement of sleeping. The pain score from the EORTC QLQ-OES18 decreased significantly from 16 to 9 (*P*=0.045), indicating less pain. The score for dry mouth increased significantly from 5 to 14 (0.005). No significant changes were seen in the other symptom scores.

From 33 patients we obtained the QLQ-C30 questionnaires at baseline and after stopping chemotherapy, from 31 patients the OES18 questionnaires at these time points. Scores on the physical functioning scale declined from 88 to 78 (*P*=0.02), but scores on the emotional functioning scale improved from 60 to 71 (*P*=0.02). From the symptom scales of the EORTC QLQ-C30, the sleeping score decreased significantly from 34 to 17 (*P*=0.03), indicating less symptoms. However, the fatigue score increased significantly from 30 to 43 (*P*=0.003), indicating more fatigue. From the symptom scales of the EORTC QLQ-OES18, the pain score decreased significantly form 15 to 7 (*P*=0.02), indicating less pain. The dry-mouth score increased significantly from 5 to 20 (*P*=0.01), indicating worsening of complaints from dry mouth. No significant changes were seen in the other symptom scores.

The global QoL score was 63 at baseline and 62 after the second course and after stopping chemotherapy.

## DISCUSSION

Our study showed no major haematological toxicity, except for grade 3 anaemia in one patient and grade 3 neutropenia in another patient. Grade 3 or 4 non-haematological toxicity was uncommon as well. However, in 22% of the patients toxicity was the reason for stopping the chemotherapy (grade 2 toxicity in seven out of 11 patients and grade 3 or 4 in four out of 11 patients). The most (in more than 85% of the patients) reported toxicities were constitutional, namely lethargy and polyneuropathy, but these were of mild to moderate intensity. Other frequently (in more than 50% of the patients) reported toxicities were gastrointestinal (nausea, vomiting, and diarrhoea), which were also generally of mild to moderate intensity. Although swallowing tablets might be a problem for patients with oesophageal cancer, all patients in this study were able to ingest the tablets.

These toxicities were comparable with that found in the study of Cassidy *et al* (2004) in patients with metastatic colorectal cancer, as were the reasons for withdrawal from therapy. In one other phase II study, the combination of oxaliplatin and capecitabine was tested as first-line treatment in patients with metastatic adenocarcinoma of the oesophagus, gastro–oesophageal junction, and gastric cardia (Jatoi *et al*, 2006). Grade 3 and 4 gastrointestinal toxicity and lethargy were more common in this study, neurological and haematological toxicities were not mentioned. The toxicity prompted a dose reduction of capecitabine to 825 mg m^−2^, because of four treatment-related deaths. An explanation for this difference in toxicity cannot be found, because the baseline characteristics of the patients enrolled in both studies seem to be comparable. However, these regional differences in mainly gastrointestinal tolerability for fluoropyrimidines between the United States and the rest of the world have been described before (Haller *et al*, 2006). In another phase II study performed in 54 patients with advanced gastric cancer (Park *et al*, 2006), the toxicity of this regimen was comparable with the toxicity in our trial.

Our study showed a median overall survival of 8 months, which is in line with the results from other studies performed at our institution in patients with metastatic or local–regional unresectable oesophageal cancer (Polee *et al*, 2003). However, the 1-year survival in our study was slightly worse compared with that observed in previous studies at our institution (26 *vs* 33%). This might be explained by the fact that in our study more patients with poor prognostic factors such as liver metastases were included (45 *vs* 23%). There were no differences in efficacy by tumour location, histology, or prior preoperative chemo(radio)therapy (results not shown). However, in view of the relatively small number of included patients, such subset analyses must be interpreted with caution.

Improving or maintaining QoL and achieving symptom relief are important goals in the management of patients with metastatic oesophageal cancer. In this study, patients reported an improvement in emotional well-being after two courses of chemotherapy as well as after stopping the chemotherapy. This is intriguing, because the physical functioning scores declined significantly over the same period. The reason for this decline in the physical functioning scores is most probably caused by the increase of fatigue, which was stated more frequently after stopping chemotherapy (median after four courses). This increase in fatigue score may be owing to the treatment, but in about half of the patients, the treatment was stopped because of disease progression, which can also lead to a higher level of fatigue. The improvement of emotional well-being during chemotherapy cannot be easily comprehended. Possibly, a better way of coping with the diagnosis of incurable cancer throughout time leads to this improvement. Second, the very act of undergoing treatment may also lead to an improvement of emotional well-being. It has been described that the QoL improves during chemotherapy, despite considerable toxicity (Cunningham *et al*, 1998). Besides, it is well known that patients are willing to undergo treatments that have small benefits with major toxicity (Matsuyama *et al*, 2006). Although the decrease of pain levels may be the effect of better pain control by the use of analgesics (opioids as well as non-opioids), it was observed that pain also decreased in patients with a stable use of analgesics. In some patients, the dosages of analgesics could be decreased or even stopped, suggesting an effect of the chemotherapy.

Overall, the global QoL score at baseline did not change over time. In the study of Ross *et al* (2002), the global QoL scores were maintained with epirubicin/cisplatin/5-fluorouracil, but declined with mitomycin/cisplatin/5-fluorouracil. In that study, the QoL scores declined over time, but the follow-up of questionnaires was longer than in our study (up to 1 year after treatment). In the study of Webb *et al* (1997), the global QoL scores were maintained and showed no difference between arms at 12 weeks *(P*=0.71), but at 24 weeks the difference became more pronounced *(P*=0.04), with the epirubicin/cisplatin/5-fluorouracil scores maintained and the 5-fluorouracil/adriamycin/methotrexate scores lowered.

In conclusion, our study shows that the activity of the combination of oxaliplatin 130 mg m^−2^ intravenously on day 1 and capecitabine 1000 mg m^−2^ orally twice daily on days 1–14 in a 21-day cycle is comparable with other chemotherapy regimens in metastatic or local–regional unresectable oesophageal cancer. The frequency of grade 3/4 toxicity was low and the QoL was maintained during the treatment. Because this treatment is probably less toxic than cisplatin-based therapy, with preservation of QoL during treatment, and because it can be given on an outpatient basis, this regimen is a viable treatment option in patients with advanced oesophageal cancer.

## Figures and Tables

**Table 1:** Baseline characteristics

	**No.**	**%**
*Gender*		
Male	44	86
Female	7	14
*Age (years)*		
Median	60	
Range	(31–76)	
*WHO*		
0	20	39
1	30	59
Missing	1	2
*Weight loss*		
<5%	17	33
5–10%	17	33
>10%	13	26
Missing	4	8
*Histology*		
Adenocarcinoma	45	88
Squamous cell carcinoma	4	8
Undifferentiated carcinoma	2	4
*Prior chemoradiotherapy/chemotherapy*		
Yes	6	12
No	45	88
*Prior surgery*		
Transhiatal oesophagectomy	17	33
Laparotomy (without resection)	4	8
*Sites of metastases*		
Lymph nodes	37	
Liver	23	
Lung	16	
Locoregional recurrence	8	
Other	8	

**Table 2:** Haematological and non-haematological toxicities

**(%)**	**Grade 0 (%)**	**Grade 1 (%)**	**Grade 2 (%)**	**Grade 3 (%)**	**Grade 4 (%)**
Anaemia	7 (14)	36 (70)	7 (14)	1 (2)	—
Leucopenia	43 (84)	6 (12)	2 (4)	—	—
Neutropenia	45 (88)	—	5 (10)	1 (2)	—
Thrombocytopenia	31 (61)	15 (29)	5 (10)	—	—
Nausea	9 (18)	29 (57)	10 (19)	3 (6)	—
Vomitus	21 (41)	20 (39)	6 (12)	4 (8)	—
Anorexia	36 (71)	10 (19)	4 (8)	1 (2)	—
Diarrhoea	23 (45)	23 (45)	4 (8)	1 (2)	—
Lethargy	6 (12)	30 (59)	12 (23)	2 (4)	1 (2)
Hand–foot syndrome	42 (82)	3 (6)	4 (8)	2 (4)	—
Polyneuropathy (sensory)	4 (8)	34 (67)	11 (21)	2 (4)	—
Polyneuropathy (motor)	47 (92)	1 (2)	1 (2)	2 (4)	—
Hyperbilirubinemia	39 (76)	8 (16)	3 (6)	1 (2)	—

Data from 51 evaluable patients.
